# Association between TERT rs2853669 polymorphism and cancer risk: A meta-analysis of 9,157 cases and 11,073 controls

**DOI:** 10.1371/journal.pone.0191560

**Published:** 2018-03-13

**Authors:** Zhengsheng Liu, Tao Wang, Zhun Wu, Kaiyan Zhang, Wei Li, Jianbin Yang, Chenxi Chen, Lei Chen, Jinchun Xing

**Affiliations:** 1 Department of Urology, The First Affiliated Hospital of Xiamen University, Xiamen, Fujian, China; 2 The First Clinical Medical College of Fujian Medical University, Fuzhou, China; 3 Zhuxi People Hospital, Hubei, China; Georgetown University, UNITED STATES

## Abstract

**Background:**

It has been reported that the functional telomerase reverse transcriptase (TERT) rs2853669 polymorphism might contribute to different types of human cancer. However, the association of this mutation with cancer remains controversial. Here, we conducted a meta-analysis to characterize this relationship.

**Materials and methods/Main results:**

A systematic search of studies on the association of TERT rs2853669 polymorphism with all types of cancer was conducted in PubMed, Embase and Cochrane Library. The summary odds ratios (ORs) and corresponding 95% confidence intervals (95% CIs) were used to pool the effect size in a fixed-effects model or a random-effects model where appropriate. A total of 13 articles and 15 case-control studies, including 9,157 cases and 11,073 controls, were included in this meta-analysis. Overall, the pooled results indicated that the rs2853669 polymorphism was significantly associated with increased cancer risk in a homozygote comparison model (CT vs. TT: OR = 1.085, 95% CI: 1.015–1.159, P = 0.016). In the stratified analyses, a significant increased cancer risk was observed in Asian, but not Caucasian patients. A subgroup analysis by cancer type also revealed a significant increase in the risk of lung cancer, but not breast cancer.

**Conclusions:**

The results of this meta-analysis suggest that the TERT rs2853669 polymorphism is associated with a significantly increased risk of cancer, particularly lung cancer, in Asian populations.

## Introduction

Telomeres are stretches of conserved, tandemly repeated DNA sequences that form the physical ends of eukaryotic chromosomes [1]. Telomeres comprise long TTAGGG nucleotide repeats with a single-stranded overhang and protein complex [2], which plays an essential role in preserving genomic stability [3] and protecting linear chromosome ends [4]. Telomeres gradually decrease in length during cell division and eventually shorten below a critical threshold, thereby triggering senescence, apoptotic cell death, or genome instability [5, 6]. Telomerase, which adds a repeat DNA sequence to the end of telomeres, is a reverse transcriptase comprising an RNA molecule and a TERT protein [1, 5]. TERT, a catalytic subunit of the enzyme telomerase, is critical for telomerase activity and has great importance in cancer progression. The activation of telomerase, which has been detected in nearly all human cancers, is a vital step in the progression of a majority of cancer types [7, 8].

Recently, many genome-wide association (GWA) studies focusing on cancer risk have been performed. TERT-locus single nucleotide polymorphisms (SNPs), located on chromosome 5p15.33, have reportedly been associated with the risk of several types of human cancers [9, 10]. A TERT promoter mutation (TERT-mut) creates a putative binding site for Ets/TCF transcription factors, increasing telomerase activity, whereas the TERT rs2853669 variant disrupts Ets/TCF binding [11]. The TERT rs2853669 T>C polymorphism (SNP), located upstream of the TERT promoter region, has been shown to affect telomerase activity and telomere length [12].

The association between TERT rs2853669 and cancer risk has since been studied in multiple ethnicities and populations with inclusive results. Thus, we performed a meta-analysis screening of all relevant published data to clarify the association between the TERT rs2853669 polymorphism and cancer risk.

## Materials and methods

### 2.1 Search strategy

Two independent investigators conducted a systematic published literature search in the PubMed, EMBASE, and Cochrane Library databases to identify studies evaluating the association between the TERT rs2853669 polymorphism and cancer risk conducted prior to October 14, 2016. The following keywords were used: “telomerase reverse transcriptase or TERT” and “cancer or tumor or carcinoma” and “polymorphism or polymorphisms or SNP or variants”. A search of all citations from the original studies was manually conducted to located further relevant studies.

### 2.2 Inclusion and exclusion criteria

The inclusion criteria for studies in the current meta-analysis were as follows: (1) designed as a case-control study evaluating the association between the TERT rs2853669 polymorphism and cancer risk; (2) studied human tissues rather than animal tissues; (3) established definitive cancer diagnosis; and (4) contained available genotype frequency information to estimate the odds ratios (ORs) and 95% confidence intervals (95% CIs). The exclusion criteria were as follows: (1) duplication of same publications; (2) reviews, letters, posters and editorials; and (3) studies lacking detailed genotype frequencies.

### 2.3 Data extraction

Two investigators independently extracted the usable data from the eligible studies, and a consensus was reached. In the case of a controversy, a consensus was reached through discussion. If the controversy remained, a third author would adjudicate the disagreements.

The following data were sought from each article: first author’s name, year of publication, country of origin, ethnicity (Caucasian, Asian and others), cancer type, source of control (hospital-based or population-based), number of cases and controls with CC, CT and CC genotypes, and Hardy Weinberg equilibrium. When Hardy Weinberg equilibrium (HWE) in the control genotype was not calculated, an online program (http://ihg.gsf.de/cgi-bin/hw/hwa1.p) was used.

### 2.4 Methodological quality assessment

Two researchers independently assessed the quality of the included studies according to the Newcastle-Ottawa Scale, of which the most significant factor was “age and gender”. The quality scores ranged from 0 to 9, and higher scores indicated better quality. The reviewers settled disagreements through discussion.

### 2.5 Statistical analysis

This meta-analysis was performed according to the checklists and guidelines by PRISMA [13]. HWE was obtained for each study using the Chi-square test in the control groups, and P<0.05 was considered a significant deviation from HWE. OR and 95% CIs were calculated to evaluate the strength of the association between the TERT rs2853669 polymorphism and susceptibility to cancer. Pooled ORs were computed from studies by allelic comparisons (C vs. T), dominant model (CC + CT vs. TT), recessive model (CC vs. CT + TT), homozygote comparisons (CC vs. TT) and heterozygote comparisons (CT vs. TT), respectively. The statistical significance level was based on a Z-test with P< 0.05.

The heterogeneity in each study was evaluated based on Cochran’s Q statistic and the I^2^ index. The random-effects model (DerSimonian and Laird method) was applied when P<0.10 and/or I^2^ index>50%. Otherwise, if the heterogeneity test value was P>0.10 and/or I^2^ index<50%, the fixed-effects model (Mantel and Haenszel method) was used. To evaluate the effect of each study on the combined ORs, a sensitivity analysis was conducted based on the leave-one-out method of each study in total and subgroups. In addition, subgroup analyses were stratified by cancer type (lung cancer, breast cancer, prostate cancer, gastric cancer, glioblastoma, colorectal cancer, acute myeloid leukemia [AML], and squamous cell carcinoma of the head and neck [SCCHN]), control type (population based and hospital based), and ethnicity (Caucasian, Asian, and others). Eventually, Begg's [14] funnel plot and Egger's [15] tests were used to statistically evaluate the publication bias. All statistical analyses were performed using Stata 12.0 software (StataCorp, College Station, Texas, USA). A two-tailed P<0.05 was considered significant except for specified conditions, where a certain P value was declared.

## Results

### 3.1 Characteristics of the studies

**Fig 1:**
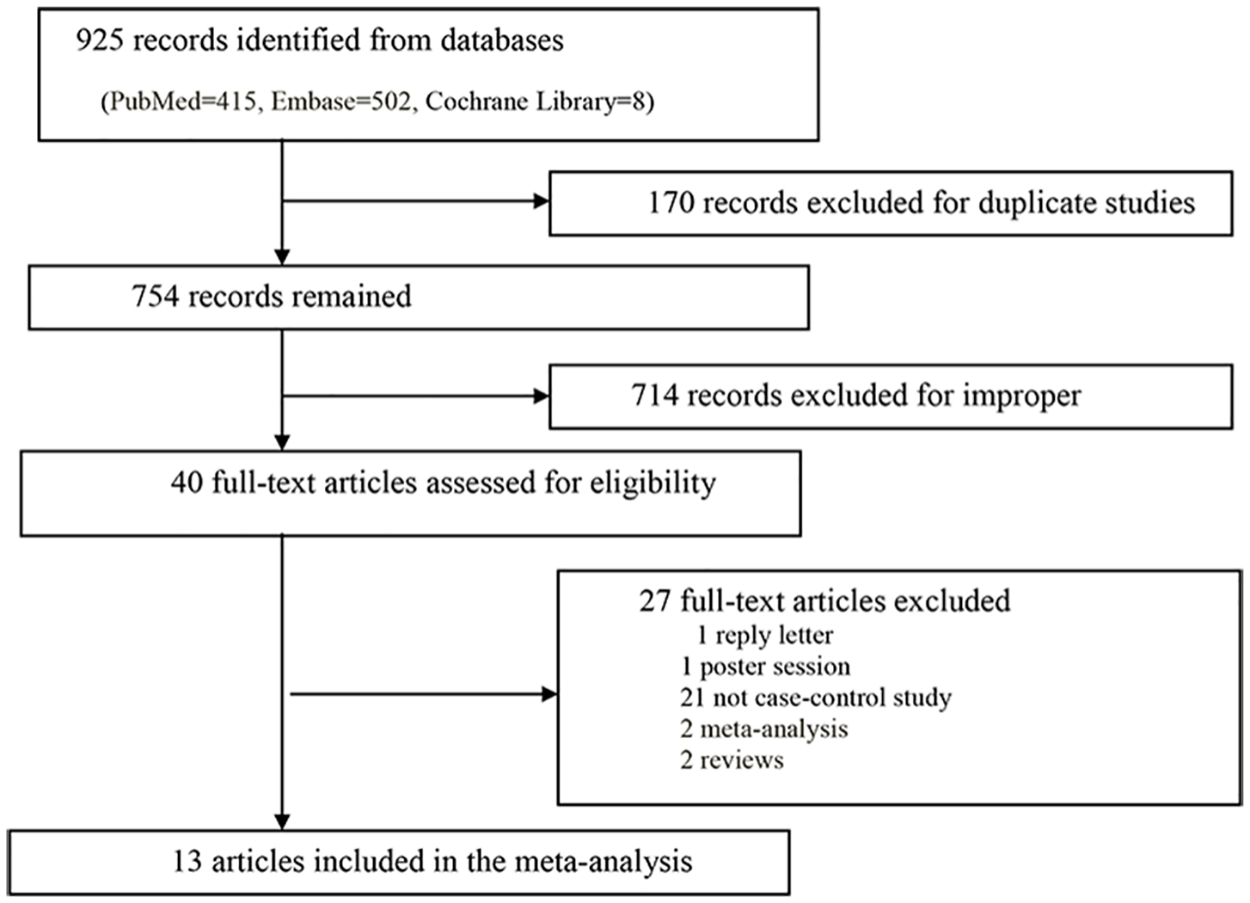
Flow chart of study selection.

A total of 925 articles were acquired from the databases (PubMed = 415, Embase = 502, Cochrane Library = 8), and thirteen articles met the inclusion criteria [16–28]. The detailed evaluation process is shown in Fig 1. Among the thirteen articles, two studies were conducted by Shadrina et al. [23] and Varadi et al. [25]. In total, fifteen case-control studies were enrolled, including 9,157 cases and 11,073 controls, to explore the relationship between the TERT rs2853669 polymorphism and cancer risk. The characteristics of the studies are shown in Table 1. Different genotyping methods were utilized, including Taqman, LightCycler (a method of real-time polymerase chain reaction), and sequencing. Blood samples were used for genotyping in all studies. Among all fifteen studies, 5 studies focused on breast cancer [21–25], three studies focused on lung cancer [26–28], and one study each focused on gastric cancer [16], prostate cancer [23], AML [20], glioblastoma [19], colorectal cancer [17] and SCCHN [18]. The cancer types were also definitively confirmed through histological or pathological analyses among all studies. Among the 15 included studies, 11 studies included Caucasian descents, three studies included Asian descents and one study included 94% Caucasian, 4% African-American and 2% other descents. The control sources were population based in 7 studies and hospital based in 8 studies.

**Table 1:** Characteristics of the studies included in the meta-analysis.

NO.	Study ID	Year	Country	Ethnicity	Cancer Type	Control Type	Cases	Controls	Case			Controls			HWE^#^	Quality^#^
									CC	CT	TT	CC	CT	TT		
1	Xing et al.	2016	China	Asian	Lung Cancer	HB*	418	410	41	162	215	30	145	235	0.249	7
2	Oztas et al.	2016	Turkey	Caucasian	Breast Cancer	HB	107	110	24	47	36	25	52	31	0.723	7
3	Bayram et al.	2016	Turkey	Caucasian	Gastric Cancer	HB	104	209	13	47	44	35	99	75	0.810	8
4	Yoo et al.	2015	Korea	Asian	Lung Cancer	HB	1100	1096	137	477	478	105	490	485	0.242	8
5	Shadrina et al.1	2014	Russian	Caucasian	Breast Cancer	HB	659	522	63	225	371	42	195	285	0.291	6
6	Shadrina et al.2	2014	Russian	Caucasian	Prostate Cancer	HB	372	363	36	169	167	30	127	206	0.104	6
7	Mosrati et al.	2015	Sweden	Caucasian	AML^#^	PB*	226	779	38	99	89	65	341	373	0.293	5
8	Mosrati et al.	2015	Sweden	Caucasian	Glioblastoma	PB	128	779	11	48	69	65	341	373	0.293	5
9	Jannuzzi et al.	2015	Turkey	Caucasian	Colorectal Cancer	HB	104	135	15	50	31	17	58	40	0.586	8
10	Zhong et al.	2013	China	Asian	Lung Cancer	PB	498	502	108	242	148	72	224	206	0.381	8
11	Liu et al.	2011	USA	Caucasian	SCCHN^#^	HB	888	885	79	381	428	85	375	425	0.863	7
12	Jing Shen al. **	2010	USA	Other***	Breast Cancer	PB	1034	1082	86	445	503	128	432	522	0.009	8
13	Varadi et al.1	2008	Poland	Caucasian	Breast Cancer	PB	768	400	58	299	411	38	154	244	0.059	7
14	Varadi et al.2**	2008	Sweden	Caucasian	Breast Cancer	PB	766	1519	47	310	409	143	558	818	0.001	7
15	Savage et al.	2007	Poland	Caucasian	Breast Cancer	PB	1985	2282	1095	766	124	1224	900	158	0.669	8

^#^: Quality was evaluated according to NOS, and the most important factor was “age and gender”.

AML: acute myeloid leukemia

SCCHN: squamous cell carcinoma of the head and neck

HWE: Hardy-Weinberg equilibrium, P< 0.05 was considered as a significant departure from HWE;

*PB: population-based; HB: hospital-based.

**P for HWE< 0.05 in controls who are all women.

***Other: 94% Caucasian, 4% African American and 2% other descent

### 3.2 Main meta-analysis results

**Fig 2:**
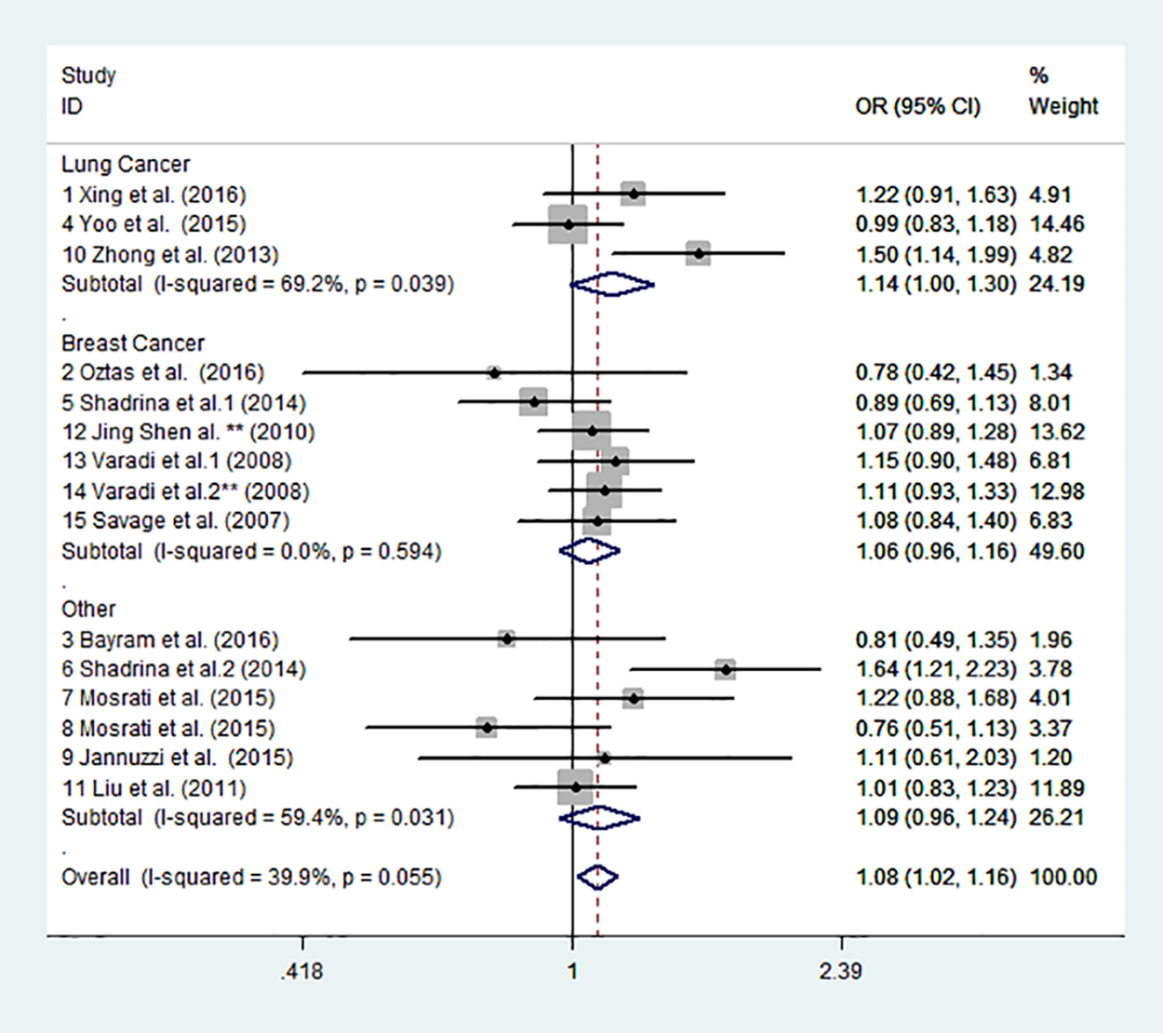
Forest plot of homozygote comparison (CT versus TT) for overall comparison, using a fixed-effects model.

According to all eligible studies, the results indicated a statistically significant association between the TERT rs2853669 polymorphism and cancer susceptibility in the homozygote comparison (CT vs. TT: OR = 1.085, 95% CI = 1.015–1.159; P = 0.016, Fig 2.) (fixed-effects model). Therefore, an association of homozygote CT with increased risk of cancer was confirmed. However, no significance was observed in the other four genetic models, including in the allele model (C vs. T: OR = 1.071, 95% CI = 0.984–1.165, P = 0.113), the homozygote comparison model (CC vs. TT: OR = 1.114, 95% CI: 0.906–1.369; P = 0.307), recessive model (CC vs. CT+TT: OR = 1.070, 95% CI = 0.903–1.268; P = 0.436) and dominant model (CT+CC vs. TT: OR = 1.098, 95% CI = 0.992–1.214; P = 0.070), whereas a trend of increased risk was observed. The results of each genetic model are shown in Table 2.

**Table 2:** Summary of pooled ORs in the stratified analysis association between TERT rs2853669 and cancer risk.

Variables	Case/control	C versus T		CT versus TT		CC versus TT		CT+CC versus TT		CC versus CT+TT	
		OR*(95% CI*)	I ^2^ (%)	OR (95% CI)	I ^2^ (%)	OR (95% CI)	I ^2^ (%)	OR (95% CI)	I ^2^ (%)	OR (95% CI)	I ^2^ (%)
Total	9157/11073	1.071(0.984–1.165)	**68.1**	**1.085(1.015–1.159)**	39.9	1.114(0.906–1.369)	**71.9**	1.098(0.992–1.214)	**55.6**	1.070(0.903–1.268)	**68.4**
Cancer type											
Breast cancer	5319/5915	0.989(0.932–1.050)	0	1.056(0.961–1.161)	0	0.880(0.703–1.102)	**53.4**	1.014(0.927–1.109)	0	0.872(0.691–1.100)	**68.4**
Lung cancer	2016/2008	**1.248(1.035–1.505)**	**71.9**	1.198(0.927–1.548)	**69.2**	**1.558(1.270–1.912)**	46.8	1.280(0.975–1.681)	**75.7**	**1.442(1.190–1.747)**	0
Others	1822/3150	1.083(0.891–1.317)	**73.2**	1.080(0.862–1.354)	**59.4**	1.180(0.789–1.764)	**68.1**	1.096(0.861–1.396)	**68.2**	1.146(0.811–1.617)	**61.2**
Source of control											
Population based	5405/7343	1.080(0.940–1.242)	**80.9**	**1.124(1.026–1.230)**	30.0	1.116(0.770–1.618)	**85.5**	1.125(0.966–1.310)	**65.2**	1.046(0.777–1.407)	**84.0**
Hospital based	3752/3730	1.066(0.994–1.143)	43.7	1.042(0.945–1.148)	**48.1**	1.149(0.981–1.346)	10.3	1.065(0.971–1.167)	50.0	1.134(0.976–1.318)	0
Ethnicity											
Caucasian	6107/7983	1.040(0.948–1.140)	**56.7**	1.068(0.981–1.162)	38.2	1.048(0.838–1.312)	**60.7**	1.060(0.978–1.148)	46.8	1.024(0.848–1.237)	**57.9**
Asian	2016/2008	**1.248(1.035–1.505)**	**71.9**	1.198(0.927–1.548)	**69.2**	**1.558(1.270–1.912)**	46.8	1.280(0.975–1.681)	**75.7**	**1.442(1.190–1.747)**	0

*PB = Population-based, HB = Hospital-based. OR = Odds ratio, CI = Confidence interval. Results with a positive significant difference are indicated in bold.

Sub-group analyses were performed to investigate the effect of cancer types, source of control and ethnicity. For cancer types, increased cancer risk was demonstrated in lung cancer in the allelic comparison (C vs. T:OR = 1.248, 95% CI = 1.035–1.505; P = 0.020), homozygote comparison (CC vs. TT: OR = 1.558, 95% CI = 1.270–1.912; P = 0.000) and recessive model (CC vs. CT+TT: OR = 1.442, 95% CI = 1.190–1.747; P = 0.000). However, no significant difference was observed in breast cancer in any genetic model (detailed in Table 2).

When stratifying according to ethnicity, greatly affected cancer susceptibility associations between the TERT rs2853669 polymorphism and cancer susceptibility were observed in Asians in the allelic comparison (C vs. T: OR = 1.248, 95% CI = 1.035–1.505; P = 0.020), homozygote comparison (CC vs. TT: OR = 1.590, 95% CI = 1.183–2.135; P = 0.000) and recessive model (CC vs. CT + TT: OR = 1.442, 95% CI = 1.190–1.746; P = 0.000), but not for Caucasians in any of the genetic models. For source of control, however, only one homozygote comparison (CT vs. TT: OR = 1.124, 95% CI = 1.026–1.230; P = 0.012) in the population-based control source showed a significant association between the TERT rs2853669 polymorphism and cancer risk.

### 3.3 Heterogeneity

As shown in Table 2, heterogeneity was observed between studies in the four genetic comparisons. Significant heterogeneity existed in the allele model (C vs. T:OR = 1.071, 95% CI = 0.984–1.165, P = 0.113), homozygote comparison model (CC vs. TT: OR = 1.114, 95% CI: 0.906–1.369; P = 0.307), recessive model (CC vs. CT+TT: OR = 1.070, 95% CI = 0.903–1.268; P_heterogeneity_ = 0.007) and dominant model (CT+CC versus TT: OR = 1.098, 95% CI = 0.992–1.214; P_heterogeneity_ = 0.005). However, when stratified based on subgroup analysis, heterogeneity disappeared in breast cancer, which was stratified into cancer type and hospital-based control source subgroups.

Meta-regression was also used to examine the source of heterogeneity based on cancer types, source of control and ethnicity in the variant heterozygote comparison (CC vs. TT). The results indicated that ethnicity (P_heterogeneity_ = 0.02) contributed to the source of heterogeneity, but not cancer types (P_heterogeneity_ = 0.15) or source of control (P_heterogeneity_ = 0.23). Consequently, ethnicity explained 44.67% of the between studies variance.

### 3.4 Sensitivity analyses

Sensitivity analysis was performed to evaluate the influence of the single studies on the pooled ORs based on the sequential removal of individual included studies each time. Subsequently, the results further demonstrated that no individual study significantly affected the pooled OR, thus, the overall results indicated the stability and reliability (not shown in figure).

### 3.5 Publication bias

**Fig 3:**
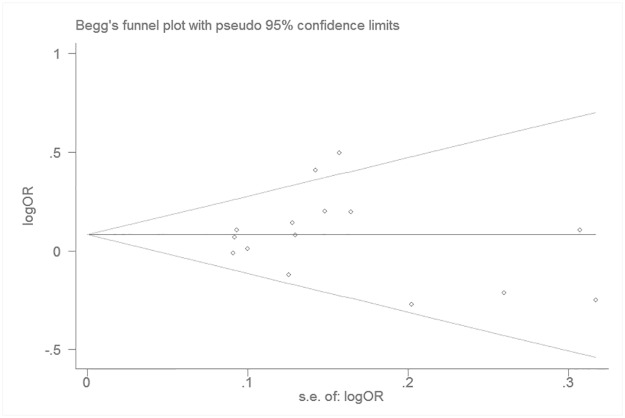
Begg’s funnel plot for publication bias analysis association between the TERT rs2853669 polymorphism and cancer risk (CT vs. TT).

Begg’s funnel plot and Egger’s test were used to estimate the publication bias of eligible studies. No publication bias for the association between TERT rs2853669 polymorphism and cancer susceptibility was manifested based on Begg’s funnel plot (P_Begg_ = 0.921, CC versus TT, Fig 3) or Egger’s regression test (P_Egger_ = 0.652, CC versus TT). Similarly, these results still did not show publication bias under the other genetic models (C vs. T P_Begg_ = 0.692, CT+CC vs. TT P_Begg_ = 0.843, CT vs. TT P_Begg_ = 0.767 and CC vs. CT + TT; P_Begg_ = 0.843). Consequently, there was no the evidence of publication bias in our meta-analysis.

## Discussion

Telomeres are structural elements that protect the ends of chromosomes from degradation, end-to-end fusion, recognition as damaged DNA and maintenance of chromosomal stability [29, 30]. TERT, encoded by the TERT gene, is an essential component of telomerase that plays a crucial role in enzymatic activity in human telomerase [31, 32]. The reactivation of telomerase drives human cell immortality, which is critical in human cancer, reflecting the fact that most cancers involve high levels of telomerase activity [33–35].

The promoter region of TERT, located at positions c.-124:C>T and c.-146:C>T, is considered a regulatory element for telomerase activity, which regulates gene transcription through several binding sites for factors [35, 36]. Moreover, the TERT promoter creates a putative binding site for Ets/TCF transcription factors to enhance telomerase expression and activity. The rs2853669 variant, a novel single nucleotide polymorphism (SNP) -245 kb upstream (Ets2 binding site) of the TERT gene, prevents Ets/TCF binding [37, 38] [39]. A recent study suggested that a functional promoter polymorphism, TERT rs2853669, may influence both telomere length and telomerase activity [27, 40]. Thus, TERT rs2853669 is strongly associated with the promoter region of telomerase reverse transcriptase. A growing number of epidemiological studies have been conducted to determine the associations between this polymorphism and cancer risk, particularly for breast cancer and lung cancer; however, the results were inconclusive.

In the present meta-analysis, 9,157 cases and 11,073 controls extracted from thirteen eligible studies were analyzed and evaluated. Significant statistics were observed for the association between the TERT rs2853669 polymorphism and cancer susceptibility in the variant CT homozygote compared with the TT wild-type homozygote model. Furthermore, three genetic models, the allelic model (C vs. T), homozygote model (CC vs. TT) and recessive model (CC vs. CT + TT), also demonstrated a strong association of increasing lung cancer susceptibility in Asian populations. Notably, no significant increasing risk of breast cancer was observed in our meta-analysis.

As shown in our meta-analysis, significant statistical heterogeneity was observed. Meta-regression was also used to detect the origin of this heterogeneity, showing that the ethnicity was the source of heterogeneity. However, when the stratification was based on subgroup analysis, heterogeneity disappeared in breast cancer, which stratified in the cancer type subgroup and hospital-based source control subgroup. Therefore, there is a strong foundation to consider between the etiology of different tumors and source of control groups, which also contributed to the source of heterogeneity. The results of the sensitivity analysis were reliable and robust. Additionally, neither the shape of the funnel plots nor the publication bias results showed statistical significance in this meta-analysis.

Surprisingly, during the subgroup analysis stratified by cancer type, no significant increase in the risk of breast cancer was observed in any genetic model. However, our result is contrary to that of Li et al. [41], who concluded that TERT rs2853669 polymorphisms were associated with the increased risk of developing breast cancer (OR = 0.76; 95% CI: 0.63–0.90; P = 0.002). Subsequently, two investigators carefully assessed the results of the meta-analysis, and a marked difference between the Li et al study and our meta-analysis was observed when studies NO.2 and NO.5 were included. When focused on lung cancer, a significant increase cancer risk was observed in association with this polymorphism. Increasing cancer susceptibility was observed in Asians, but not for Caucasians, when stratified by ethnicity. Consistently, three lung cancer studies [26–28] were conducted in Asians, thus an increased risk was observed in the allelic comparison (C vs. T), homozygote comparison (CC vs. TT) and recessive model (CC vs. CT+TT). This finding suggests that different populations might have different tumorigenesis mechanisms. Similarly, in the subgroup analysis stratified by source of control, significant associations were observed in the population-based studies, but not in the hospital-based studies.

In addition, the potential limitations of our meta-analysis should also be addressed. First, insufficient published studies were included in this meta-analysis, and more individual studies were required to determine a precise conclusion. Second, the results of gene-to-environment interactions were not obtained because of a lack of relevant information. Third, studies NO.12 and NO.14 did not meet the expectations of Hardy-Weinberg equilibrium, as both studies were focused on breast cancer. Fourth, in our meta-analysis, one cancer type (lung cancer or breast cancer) was only focused on one ethnicity (Asian or Caucasian). Therefore, additional high-level studies on different ethnicities are still needed.

## Conclusions

In conclusion, this meta-analysis suggests that the TERT genetic polymorphism rs2853669 is associated with an increased risk of cancer, particularly for lung cancer among Asians, while there is no significant association with increased risk of breast cancer. However, more functional studies with additional subgroups should be conducted to validate our findings.

## Supporting information

S1 File Meta-analysis on genetic association studies checklist. (DOCX)

S2 File PRISMA checklist. (DOCX)
